# High-sensitivity C-reactive protein among people living with HIV on highly active antiretroviral therapy: a systemic review and meta-analysis

**DOI:** 10.1186/s12879-024-09050-4

**Published:** 2024-02-02

**Authors:** Sihle E. Mabhida, Zandile J. Mchiza, Kabelo Mokgalaboni, Sidney Hanser, Joel Choshi, Haskly Mokoena, Khanyisani Ziqubu, Charity Masilela, Bongani B. Nkambule, Duduzile E. Ndwandwe, André P. Kengne, Phiwayinkosi V. Dludla

**Affiliations:** 1 Non-Communicable Diseases Research Unit, South African Medical Research Council, Tygerberg, 7505 South Africa; 2 School of Public Health, University of the Western Cape, Bellville, 7535 South Africa; 3 Department of Life and Consumer Sciences, University of South Africa, Roodepoort, 1709 South Africa; 4 Department of Physiology and Environmental Health, University of Limpopo, Sovenga, 0727 South Africa; 5 Department of Biochemistry, North-West University, Mmabatho, 2745 South Africa; 6 School of Laboratory Medicine and Medical Sciences, University of KwaZulu-Natal, Durban, 4000 South Africa; 7 Cochrane South Africa, South African Medical Research Council, Tygerberg, 7505 South Africa; 8 Department of Medicine, University of Cape Town, Cape Town, 7700 South Africa; 9 Department of Biochemistry and Microbiology, University of Zululand, KwaDlangezwa, Richards Bay, 3880 South Africa

**Keywords:** High-sensitivity C-reactive protein, Inflammation, Cardiovascular disease risk, Human immunodeficiency virus, Highly active antiretroviral therapy

## Abstract

The pathological consequences of inflammation persist in people living with the human immunodeficiency virus (PLWH), regardless of the positive outcomes of highly active antiretroviral therapy (HAART). The current systematic review and meta-analysis aims to understand and explore the levels of high-sensitivity C-reactive protein (hs-CRP) and other cardiovascular disease (CVD)-risk factors including lipid profiles among PLWH on HAART. Major electronic databases including PubMed, Scopus, and Web of Science were searched to retrieve relevant global literature reporting on hs-CRP levels in PLWH on HAART. A total of twenty-two studies with an average participant age of 40 years were eligible for this systematic review and meta-analysis. Majority of the included studies were from Africa (*n* = 11), the United States (*n* = 6), and Europe (*n* = 5). Our systemic review showed that most studies reported increased levels of hs-CRP among PLWH on HAART when compared to controls (PLWH not on HAART or those without HIV), especially in studies from Africa. This was supported by a meta-analysis showing significantly elevated levels of hs-CRP in PLWH on HAART when compared to PLWH not on HAART (standardised mean difference [SMD] = 0.56; 95% CI = 0.10‑1.01, z = 2.41; *p* = 0.02) or those without HIV (SMD = 1.19; 95% CI = 0.76‑1.63, z = 5.35; *p *< 0.001). Where lipid profiles, as a major predictor for CVD risk, were also impaired in PLWH on HAART when compared to PLWH not on HAART and HIV-negative participants. In conclusion, elevated levels of hs-CRP and lipid levels are prevalent in PLWH on HAART, this may increase the risk of CVD complications, especially for those people living in Africa. However, more evidence in larger population studies is required to confirm these outcomes and unveil any possible clinical implications of HAART-induced modulation of hs-CRP levels in PLWH.

**Supplementary Information:**

The online version contains supplementary material available at 10.1186/s12879-024-09050-4.

## Introduction

The human immunodeficiency virus (HIV) is a persistent public health problem that currently affects approximately 36.9 million individuals worldwide [1]. This pandemic has grave economic implications, especially in high-prevalence regions such as sub-Saharan Africa (SSA) [2]. Sub-Saharan Africa remains the epicentre for the HIV pandemic, accounting for more than 70% of the global infected population [3]. However, the availability of highly-active antiretroviral therapy (HAART) has been widely acknowledged for its effectiveness in significantly improving life expectancy and quality of life among people living with HIV (PLWH) [4–6]. Despite these improvements, prolonged use of HAART has been associated with other comorbidities (diabetes mellitus, hypertension, and dyslipidemia) that may lead to the development of cardiovascular diseases (CVDs) [7, 8]. Noteworthy, PLWH on long-term HAART are predicted to be two times more likely to develop CVDs compared to those without this condition [9], whilst the proportion of deaths attributed to CVDs in PLWH on HAART has doubled in the last decade [6, 8]. This knowledge has shifted the focus of care for PLWH, and it now calls for a better understanding of the disease pathophysiology, which makes it necessary to devise new intervention strategies to combat CVD-related complications.

Traditional risk factors and comorbidities such as diabetes mellitus, hypertension, and dyslipidemia are known to be associated with the development and progression of CVDs [10, 11]. These factors are often accompanied by inflammation, a pathological hallmark for HIV infection and CVDs [12]. Although inflammation is necessary for an adequate immune response, a dynamic balance must be achieved between pro- and anti-inflammatory factors in order to suppress infection and minimize any metabolic complications [13, 14]. Beyond their involvement in driving undesired immune activation [15], the most commonly studied pro-inflammatory markers with regard to the pathogenesis of HIV and CVD include interleukin 6 (IL-6), tumor necrosis factor (TNF-α), and high-sensitivity C-reactive protein (hs-CRP). These markers are, in part, associated with the extended use of HAART [16, 17]. Having previously been considered a traditional marker of infection and cardiovascular events [18], hs-CRP is now acknowledged for its role in the underlying inflammatory processes. This includes its activation of other pro-inflammatory cytokines such IL-6 and TNF-α [19].

As such, persistently elevated levels of hs-CRP are deemed to be among the reliable predictors of CVDs in PLWH on HAART [20–25]. However, other researchers have not seen this effect in PLWH on HAART [26, 27]. Several factors can contribute to the negative association in PLWH, including high lipid profiles, ethnicity, geographical location, and duration of treatment with HAART [24, 26]. Besides updating the status of clinical evidence on the role of this pro-inflammatory marker in PLWH, it remains essential to establish or generate data to evaluate whether hs-CRP levels may be a reliable biomarker to predict CVD risk in PLWH on HAART. Perhaps highlighting the significance of the current systematic review and meta-analysis to assess the levels of hs-CRP in relation to the manifestation of CVDs in PLWH on HAART.

## Methods

### Search strategy

A complete global literature search for publications dating from 1996 (after the introduction of HAART [28]) until August 2023 was conducted using medical subject headings (MeSH) including “C-reactive protein”, “CRP”, “cardiovascular disease”, “CVD”, “human immunodeficiency virus”, “HIV”, “inflammation”, “ART”, “antiretroviral therapy” and “highly active antiretroviral therapy” following the Preferred Reporting Items for Systematic Reviews and Meta-Analyses (PRISMA) 2020 guidelines [29]. The search strategy that was applied is attached as Table S1. The search was done thoroughly by two independent investigators (SEM and KM). Databases searched included PubMed, Scopus, and Web of Science to identify all relevant articles. A manual search on Google Scholar was also done to identify any extra studies and to identify grey literature, especially data from conference proceedings. The meta-analysis was not registered with the Prospective Register of Systematic Reviews (PROSPERO), however caution was taken not to duplicate any existing systematic review and meta-analysis on hs-CRP.

### Inclusion criteria and data extraction

Studies were included if they met the following criteria: (a) observational studies and clinical trials; (b) evaluated the modulation of the inflammatory marker hs-CRP in PLWH on HAART. Studies were excluded if (a) they were conducted before the introduction of HAART (1996), (b) they were nonhuman studies or (c) reviews. The current review and meta-analysis applied the following PECO (population, exposure, control, and outcomes):

- ***Participants*:** PLWH on HAART.
- ***Exposure*:** PLWH receiving any form of HAART regimen.
- ***Control*:** PLWH not on HAART and HIV-negative participants.
- ***Outcome*:** Hs-CRP levels and CVD-related outcomes.

### Data extraction

The extracted data was independently and carefully assessed for compliance with the inclusion or exclusion criteria by three authors who resolved disagreements by consensus. The following information was extracted from each study: the first author, publication year, country, ethnicity, sample size, mean age, treatment duration, and key findings. Language restrictions were not applied during the search, however, studies conducted in other languages that could not be translated into English were excluded. The American Heart Association and Centre for Disease Control classification of cardiovascular risk according to hs-CRP level were used. For example, a hs-CRP level of > 3mg/L represents a high risk, 1-3mg/L intermediate risk, and < 1 mg/L low risk for CVD in humans [25, 30].

### Quality assessment

For studies incorporated in the current systematic review and meta-analysis, the quality of evidence and risk of bias assessment was evaluated using the modified Downs and Black checklist, which rates studies out of 27 questions [31]. The Downs and Black checklist assesses five domains to determine the quality of the study, this includes: reporting bias, external validity, internal validity, selection bias, and power. The quality of evidence and risk of bias was based on evidence reported in the full-text article deemed eligible for inclusion in this systematic review and meta-analysis. Two independent investigators assessed the quality of evidence and the risk of bias for the eligible studies. Disagreements among investigators were resolved by consulting a third independent investigator.

### Statistical analysis

The effect size for continuous data across all outcomes was determined by calculating the mean, standard deviation (SD), and sample size for each study. In instances where the included study only reported the mean, the SD was computed using the interquartile range (IQR) [32]. The meta-analysis results are presented as forest plots for all outcome measures, and the pooled Standard Mean Difference (SMD) was calculated using the random effects model meta-analysis. Statistical analysis considered I^2^ < 25%, 25–75%, and > 75% for minimal, moderate, and extreme heterogeneity, respectively [33]. All analyses were performed using Review Manager version 5.4.1 (The Nordic Cochrane Centre, The Cochrane Collaboration, 2020).

## Results

### Characteristics of included studies

**Fig. 1:**
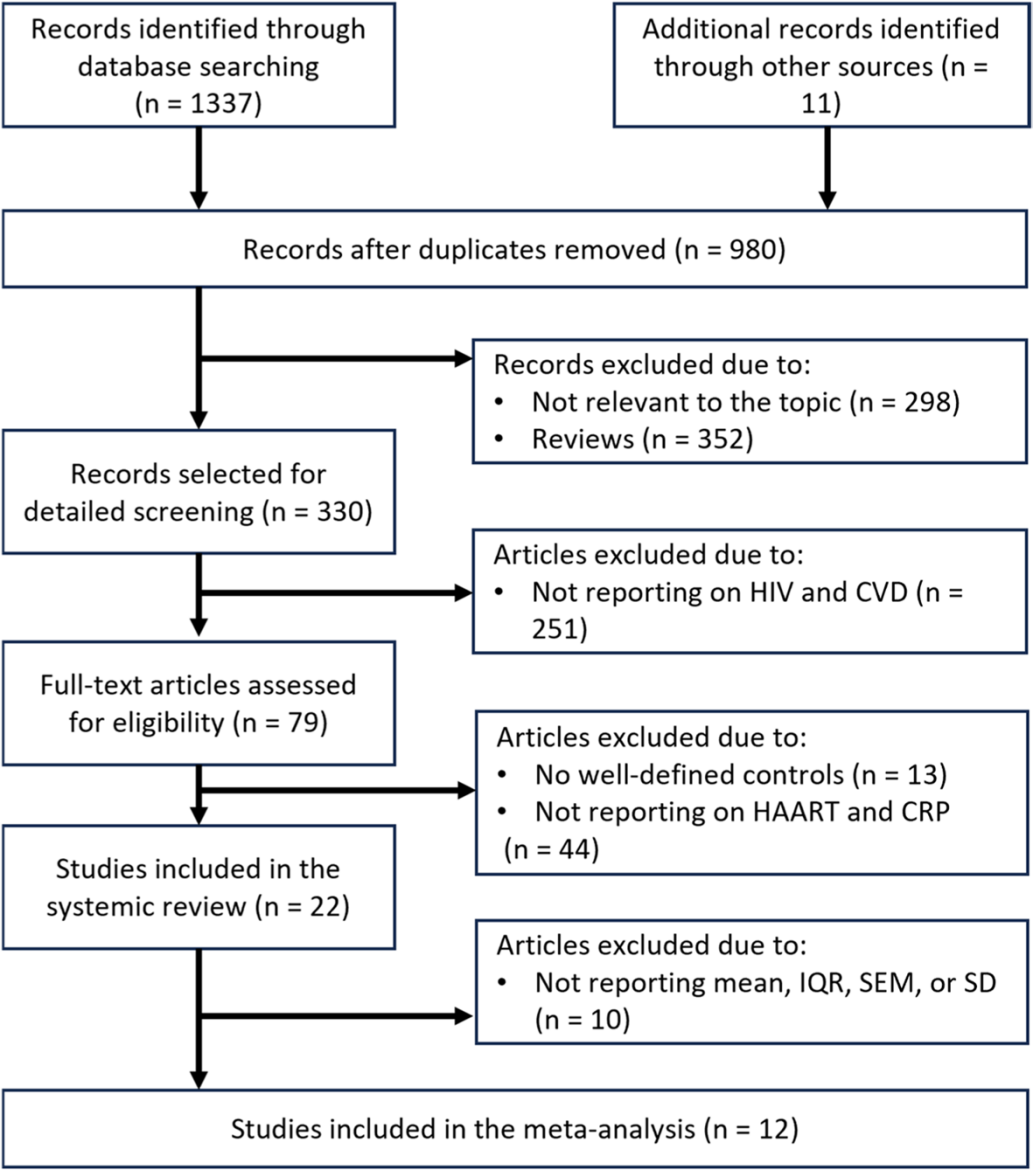
The flow diagram presents study selection. Briefly, the preliminary search of major electronic databases identified a total of 1009 articles, however only 22 of those were included in the qualitative analysis, and 12 in the meta-analysis

Our systematic search strategy identified a total of 1337 relevant records (Fig. 1). However, the final screening process yielded 22 eligible publications, that reported on hs-CRP levels in PLWH on HAART. In terms of region, most included studies were from Africa (*n* = 11), followed by the United States (*n* = 6), whilst the remaining literature was from Europe (*n* = 5). Included studies were published between the years 2004 to 2020, with the included participants having an average of 40 years. Included studies contained varied sample sizes for PLWH on HAART, ranging from the highest (*n* = 170) monitored for 9 months [34], and the smallest number of PLWH on HAART (*n* = 19) monitored for 24 months [35]. The majority of studies in the review used blood serum samples (*n* = 16) rather than plasma (*n* = 6) to analyze the hs-CRP levels.

### Qualitative analysis of included literature

The included studies were predominantly from the African continent, especially countries from sub-Saharan Africa. Furthermore, most of the studies (*n* = 11) showed that serum levels of hs-CRP were significantly elevated in PLWH on HAART (Table 1). Some of these studies indicated that hs-CRP may occur concurrently with other CVD-related complications as elevated levels of this pro-inflammatory maker were consistent with increased systolic blood pressure and abnormal lipid profiles [26, 36]. Increased systolic blood pressure [36] and abnormal lipid profiles, including elevated levels of total cholesterol (TC), low-density lipoprotein (LDL)-c, and triglycerides are well-established indicators of CVD-risk [37, 38]. It was interesting to see that waist circumference [39], and other pro-inflammatory markers such as tumor necrosis factor (TNF)-α were persistently high in PLWH on HAART [40], in studies based in Africa. In fact, it was interesting to observe that hs-CRP levels remained persistently high in PLWH taking the preferred first-line HAART regimen (Stavudine: d4T, Lamivudine: 3TC, and Efavirenz: EFZ) [41] regardless of study duration. This likely indicates that immune activation and inflammation persist in PLWH regardless of viral suppression, as previously discussed [42]. Also disputing the fact that ethnicity and environmental factors could play a major role in driving these pathological features, as some studies from the United States (*n* = 6) and Europe (*n* = 5) showed conflicting results in terms of hs-CRP levels and coronary heart disease (CHD) outcomes in PLWH on HAART when compared to those from Africa.

**Table 1:** An overview of clinical studies reporting on the levels of C-reactive protein, together with cardiovascular disease related outcomes, in people living with human immunodeficiency virus (PLWH) on high active antiretroviral therapy (HAART)

Year, Reference	Country, Ethnicity	Study population, including age and exposure period	Type of HAART and exposure period	Key findings
**Studies from Africa**				
Mutevedzi et al., 2013 [43]	South Africa, Black Africans	PLWH on HAART (*n* = 108), with an average of 57 years	Type of HAART regimen not disclosed, but participants were monitored for 12 months	Serum levels of hs-CRP levels were significantly increased in the study population
Botha et al., 2014 [36]	South Africa, Black Africans	PLWH on HAART (*n* = 66), with an average of 48 years	Received a combination of d4T, 3TC, and EFZ, and were monitored for 36 months	Serum levels of hs-CRP were higher, and this was accompanied by increased CVD-related outcomes, including systolic blood pressure, as well as TC, LDL-c, and TG
Canipe et al., 2014 [44]	Zambia, Black Africans	PLWH on HAART (*n* = 33), with an average of 36 years	Received a combination of d4T, 3TC, and EFZ and were monitored for 3 months	Serum hs-CRP were elevated, and this was correlated with BMI
Ssinabulya et al., 2014 [45]	Uganda, Black Africans	PLWH on HAART (*n* = 34), with an average of 37 years	Received a combination of d4T, 3TC, NVP; ZDV to TDF, 3TC, EFZ] or NVP, and were monitored for 60 months	Serum levels of hs-CRP and this was positively correlated with high waist circumference, TG, and HDL: LDL-c ratio
Fourie et al., 2015 [38]	South Africa, Black Africans	PLWH on HAART (*n* = 66), with an average of 49 years	Received a combination of d4T/3TC/EFV (or NVP) and monitored for 60 months	Serum levels of hs-CRP, together with IL-6 were not affected. However, lipid profiles were abnormal indicating increased TC, LDL-c, and reduced HDL-c levels in these individuals
Gleason et al., 2015 [46]	Ethiopia, Black Africans (Amhara)	PLWH on HAART (*n* = 91), with an average of 39 years	Received a combination of TDF (or AZT)/3TC/EFV; AZT (or TDF)/3TC/NVP; AZT (or TDF)/3TC/LPV-r; or ddI/ABC (or d4T)/LPV-r and monitored for 60 months	Serum levels if hs-CRP levels were increased, and this correlated with high levels of TG, LDL, including elevated heart rate
Zhou et al., 2016 [34]	Zimbabwe, Black Africans	PLWH on HAART (*n* = 170), with an average of 41 years	Received a combination of NVP/3TC/TDF, EFV/3TC/TDF, or ZDV/3TC/TDC for 9 months	Serum levels of hs-CRP were significantly in the study population
Borkum et al., 2017 [47]	South Africa, Black Africans	PLWH on HAART (*n* = 46), with an average of 42 years	Received a combination of d4T/3TC/EFV (or NVP); or AZT/ddI/LPV-r, and monitored for 90 months	Serum levels of hs-CRP were significantly increased, and this was accompanied by a high waist circumference
Muswe et al., 2017 [48]	Zimbabwe, Black Africans	PLWH on HAART (*n* = 124), with an average of 42 years	Received a combination of TDF/NVP/3TC; TDF/EFV/ 3TC; STV (or ZDV)/NVP/3TC; TDF/ATV/RTV; ABC/ATV/3TC; or TDF + ATV + 3TC. Participants were monitored for 120 months	Plasma levels of hs-CRP, together with TNF-α were significantly high in the study population
Appiah et al., 2020 [27]	Ghana, Black Africans	PLWH on HAART (*n* = 156), with an average of 48 years	TDF + 3TC + EFZ; AZT + 3TC + NVP; AZT + 3TC + EFZ; TDF + 3TC + LPV/r, TDF + 3TC + NVP; AZT + 3TC; AZT + NVP. Participants were monitored for 16 months	Serum levels of hs-CRP were significantly high, and this was correlated with elevated TG levels and high waist circumference ratio
Bestawros et al., 2015 [17]	Zambia, Black Africans	PLWH on HAART (*n* = 33), with an average of 36 years	Received a combination of EFV/TDF/FTC and were monitored for 3 months	Serum levels of hs-CRP, together with TNF-α were elevated
**Studies from the United States**				
Hurwitz et al., 2004 [49]	United States, African American and Caucasians	PLWH on HAART (*n* = 41), with an average of 41 years	Type of HAART regimen not disclosed, but participants were monitored for 6 months	Serum levels of hs-CRP were not significantly affected
Boger et al., 2009 [50]	United States, Caucasians	PLWH on HAART (*n* = 19), with an average of 44 years	Received 35% were on two NRTIs plus TFV, 33% on ZDV, 22% on ABC, 9% on ddI, and 7% on d4T. 40% were on a PI, 33% were on a NNRTI, and 2% were on ENF, and this was for 24 weeks	Serum levels of hs-CRP were elevated, and this was linked high BMI, as well as impaired lipid profiles, including TG and lower HDL-c
Ticona et al., 2015 [51]	United States, Peruvians	PLWH on HAART (*n* = 49), with an average of 37 years	Type of HAART regimen not disclosed, but participants were monitored for 24 months	PLWH on HAART had elevated plasma hs-CRP and IL-6 levels
Hileman et al., 2013 [52]	United States, African American and Caucasians	PLWH on HAART (*n* = 36), with an average of 48 years	Received ATV-containing regimen, and were monitored for 3 months	Plasma hs-CRP and IL-8 levels were elevated in studied population
Desvarieux et al., 2013 [53]	United States, Black Africans, Indians and Caucasian	PLWH on HAART (*n* = 50), with an average of 41 years	Type of HAART regimen not disclosed, but participants were monitored for 48 months	Serum levels of hs-CRP were not significantly affected
Syed et al., 2013 [54]	United States, African American and Caucasian	PLWH on HAART (*n* = 67), with an average of 17 years	Type of HAART regimen not disclosed, but participants were monitored for 12 months	Plasma levels of hs-CRP were not significantly affected, although concentrations of TG, VLDL, and TC were raised
**Studies from Europe**				
Calmy et al., 2009 [55]	Switzerland, Asian	PLWH on HAART (*n* = 34), with an average of 42 years	Received a combination of SQV/r and d4T/ddI, TDF/3TC or TDF/FTC, and monitored for 3 months	Serum levels of hs-CRP were not significantly affected in the study population
Padilla et al., 2011 [56]	Spain, Asian	PLWH on HAART (*n* = 50), with an average of 37 years	Received PI or NNRTI, and monitored for 12 months	Plasma levels of hs-CRP were not significantly affected in the study population
Ghislain et al., 2015 [57]	France, Black African	PLWH on HAART (*n* = 208), with an average of 38 years	Received a combination of 2 NRTI + 1 PI/r; 2 NRTI + 1 NNRTI, and monitored for 36 months	Serum levels of hs-CRP were reduced in the study population
Goedel et al., 2019 [26]	Germany, Caucasian	PLWH on HAART (*n* = 48), with an average of 64 years	Type of HAART regimen not disclosed, but participants were monitored for 60 months	Serum levels of hs-CRP were not significantly affected in the study population
Di Yacovo et al., 2020 [35]	Spain, Asian	PLWH on HAART (*n* = 31), with an average of 37 years	Received a combination of ABC/3TC (or FTC)/EFV; TDF/FTC/NVP; ABC/3TC (or FTC)/ATV-r (or DRV-r, LPV-r); or TDF/FTC/RAL. Participants were monitored for 24 months	Plasma levels of hs-CRP were significantly high, and this was correlated with raised concentrations of TC, LDL-c, TC/HDL-c, and TG

*Abbreviations: NRTIs* Nucleoside/nucleotide reverse transcriptase inhibitors, *3TC* Lamivudine, *ABC* Abacavir, *ATV* Atazanavir, *BIC* Bictegravir, *CAB* Cabotegravir, *COBI or /c* Cobicistat, *d4T* Stavudine, *ddI* Didanosine, *DMPA* Depot medroxyprogesterone acetate, *DOR* Doravirine, *DRV* Darunavir, *DTG* Dolutegravir, *EFV* Efavirenz, *ETR* Etravirine, *EVG* Elvitegravir, *FPV* Fosamprenavir, *FTC* Emtricitabine, *FTR* Fostemsavir, *IBA* Ibalizumab, *IDV* Indinavir, *LPV* Lopinavir, *MVC* Maraviroc, *NFV* Nelfinavir, *NVP* Nevirapine, *RAL* Raltegravir, *RPV* Rilpivirine, *RTV or /r* Ritonavir, *SQV* Saquinavir, *T-20* Enfuvirtide, *TAF* Tenofovir alafenamide, *TDF* Tenofovir disoproxil fumarate, *TFV* Tenofovir, *TFV-DP* Tenofovir diphosphate, *Tris* Tris(hydroxymethyl)aminomethane, *TMP-SMX* Trimethoprim sulfamethoxazole, *TMR* Temsavir, *TPV* Tipranavir, *ZDV* Zidovudine, *EFZ* Efavirenz, *VLDL* Very low-density lipoprotein, *TC* Total cholesterol, *TG* Triglycerides, *HDL* High density lipoprotein cholesterol, *LDL* Low density lipoprotein cholesterol, *hs-CRP* High sensitivity C-reactive protein, *TNF-α* Tumor necrosis factor, *IL-6* Interleukin, *IL-8* Interleukin, *BMI* Body mass index, *CVD* Cardiovascular diseases, *PLWH* Human immunodeficiency virus, *HAART* High active antiretroviral therapy

### Quantitative analysis of included literature

#### Circulating levels of hs-CRP in PLWH on HAART in comparison to PLWH not on HAART

**Fig. 2:**
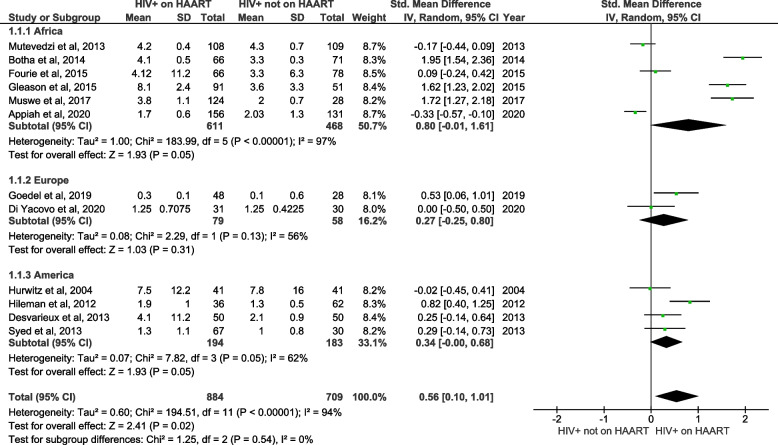
A forest plot showing outcomes of a meta-analysis for high sensitivity C-reactive protein (hs-CRP) levels in people living with the human immunodeficiency virus (PLWH) on highly active antiretroviral therapy (HAART), compared to PLWH not on HAART. Green squares represent the weight of each study in the average effect size. Horizontal lines across green squares represent the 95% confidence intervals for the point estimate. The diamonds represent the weighted average point estimate

Firstly, we analyzed the levels of hs-CRP in PLWH on HAART versus PLWH not on HAART (Fig. 2). The quantitative (pooled) analysis of twelve included studies showed that hs-CRP levels were significantly increased in PLWH on HAART when compared to PLWH not on HAART (SMD = 0.56; 95% CI = 0.10 ‑1.01, z = 2.41; *p* = 0.02). The subgroup analysis, based on the region where the study was performed, revealed no association; however, elevated levels of hs-CRP favoured studies from Africa (SMD = 0.80; 95% CI = -0.01‑ 1.61, z = 1.93; *p* = 0.05), Europe (SMD = 0.27; 95% CI = -0.25 ‑ 0.80, z = 1.03; *p* = 0.31) and the United States (SMD = 0.34; 95% CI = -0.00 ‑ 0.68, z = 1.93; *p* = 0.05). However, the tests for subgroup differences showed no statistically significant differences (*p* = 0.54) with 0% of heterogeneity (Fig. 2). Suggesting that geographical region does not influence hs-CRP levels in PLWH on HAART versus PLWH not on HAART.

#### Circulating levels of hs-CRP in PLWH on HAART in comparison to individuals without HIV

**Fig. 3:**
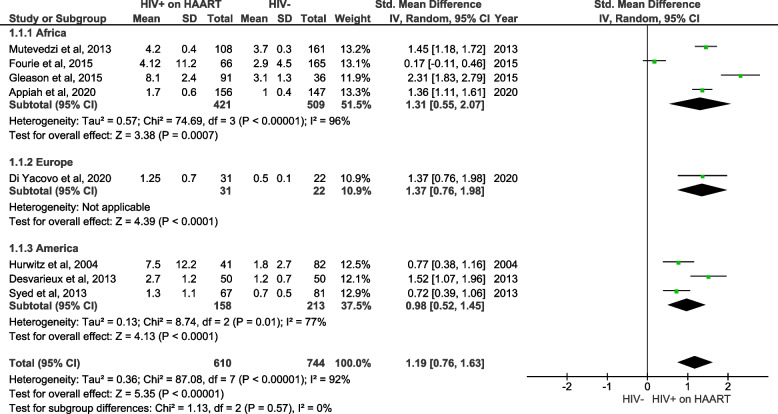
A forest plot showing outcomes of a meta-analysis for high sensitivity C-reactive protein (hs-CRP) levels in people living with the human immunodeficiency virus (PLWH) on high active antiretroviral therapy (HAART), compared to HIV-negative controls. Green squares represent the weight of each study in the average effect size. Horizontal lines across green squares represent the 95% confidence intervals for the point estimate. The diamonds represent the weighted average point estimate

The second aim of this meta-analysis, utilizing pooled data, was to evaluate whether HAART treatment affects hs-CRP levels in PLWH when compared to uninfected individuals (Fig. 3). The quantitative (pooled) analysis of eight included studies showed that hs-CRP levels were significantly increased in PLWH on HAART, in comparison to individuals without HIV (SMD = 1.19; 95% CI = 0.76 ‑ 1.63, z = 5.35; *p* < 0.001). The performed subgroup analysis showed no statistical significance between subgroups (*p* = 0.57) with 0% heterogeneity. Suggesting that geographical region does not influence hs-CRP levels in PLWH on HAART versus HIV-negative control. However, there was a significant increase in hs-CRP levels in PLWH on HAART when compared to negative controls in studies from Africa (SMD = 1.31; 95% CI = 0.55 ‑ 2.07, z = 3.38; *p* = 0.0007), Europe (SMD = 1.37; 95% CI = 0.76 ‑ 1.98, z = 4.39; *p* < 0.0001) and United States (SMD = 0.98; 95% CI = 0.52 ‑ 1.45, z = 4.13; *p* < 0.001).

#### Circulating levels of hs-CRP in PLWH not on HAART in comparison to individuals without HIV

**Fig. 4:**
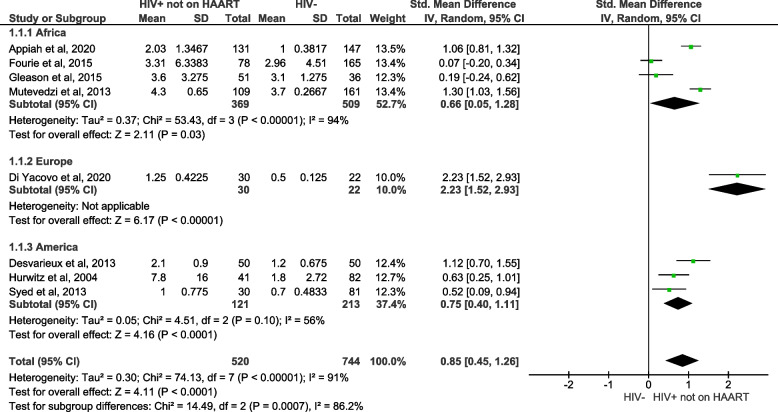
A forest plot shows meta-analysis outcomes for high sensitive C-reactive protein (hs-CRP) levels in people living with the human immunodeficiency virus (PLWH) not on highly active antiretroviral therapy (HAART), compared to HIV-negative controls. Green squares represent the weight of each study in the average effect size. Horizontal lines across green squares represent the 95% confidence intervals for the point estimate. The diamonds represent the weighted average point estimate

The third objective of this meta-analysis of pooled data was to assess whether HIV infection increases the levels of hs-CRP in PLWH not on HAART when compared with uninfected individuals (Fig. 4). The overall analysis showed that hs-CRP levels were significantly increased in PLWH not on HAART, in comparison to individuals without HIV (SMD = 0.85; 95% CI = 0.45‑1.26, z = 4.11; *p* < 0.0001). There was a significant association between PLWH not on HAART and elevated levels of hs-CRP in studies conducted in Africa (SMD = 0.66; 95% CI = 0.05‑1.28, z = 2.11; *p* = 0.03), Europe (SMD = 2.23; 95% CI = 1.52‑2.93, z = 6.17; *p* < 0.0001) and the United States (SMD = 0.75; 95% CI = 0.40‑1.11, z = 4.16; *p* < 0.0001).

#### Lipid profiles in PLWH on HAART in comparison to PLWH not on HAART

**Fig. 5:**
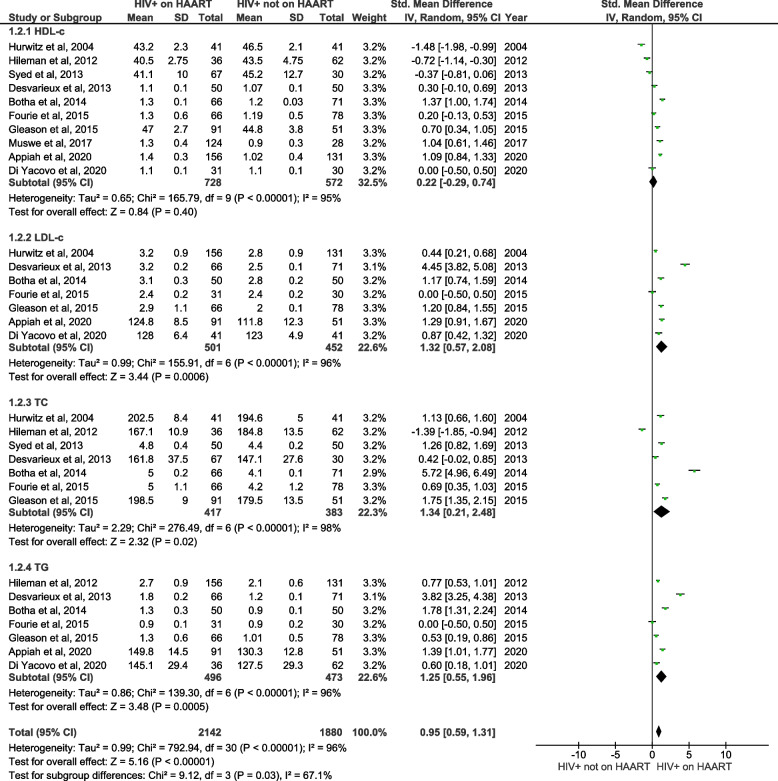
A forest plot showing meta-analysis outcomes for lipid profiles, including high density lipoprotein-cholesterol (HDL-c), low density lipoprotein-cholesterol (LDL-c), total cholesterol (TC) and triglycerides (TG), in people living with the human immunodeficiency virus (PLWH) on highly active antiretroviral therapy (HAART) in comparison to PLWH not on HAART. Green squares represent the weight of each study in the average effect size. Horizontal lines across red squares represent the 95% confidence intervals for the point estimate. The diamonds represent the weighted average point estimate

We conducted an analysis of lipid profile levels, encompassing of HDL-c, LDL-c, total cholesterol, and triglycerides, in PLWH on HAART in comparison to PLWH not on HAART (Fig. 5). Specific analyses revealed a non-significant effect on HDL-c (SMD = 0.22; 95% CI = -0.29 ‑ 0.74, z = 0.84; *p* = 0.40), but significantly increased LDL-c (SMD = 1.32; 95% CI = 0.57 ‑ 2.08, z = 3.44; *p* = 0.0006), total cholesterol (SMD = 1.34; 95% CI = 0.21 ‑ 2.48, z = 2.32; *p* = 0.02), and triglyceride (SMD = 1.25; 95% CI = 0.55—1.96 z = 3.48; *p* < 0.0005) levels in PLWH on HAART compared to PLWH not on HAART. Notably, there was a substantial level of heterogeneity, exceeding 95%, in these lipid profiles.

#### Lipid profiles in PLWH on HAART in comparison to individuals without HIV

**Fig. 6:**
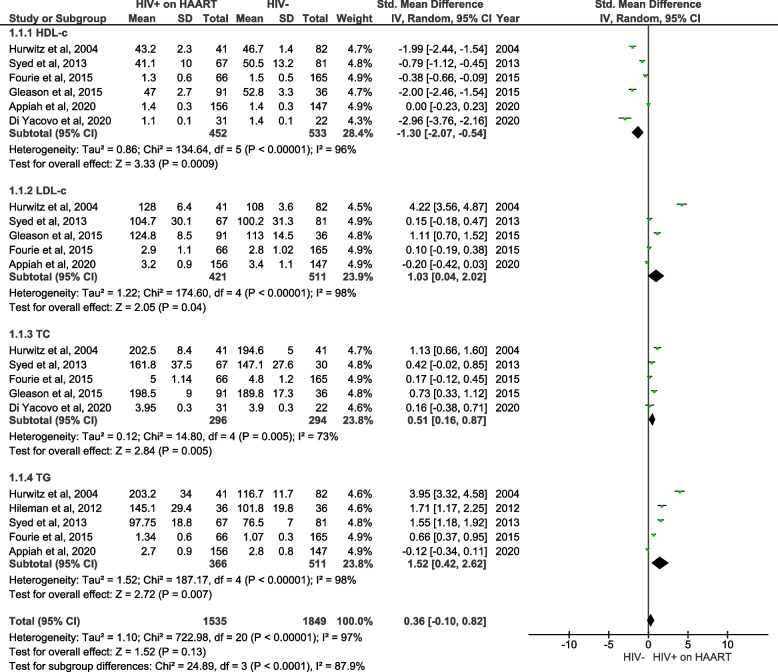
A forest plot showing outcomes of a meta-analysis for lipid profiles, including high density lipoprotein-cholesterol (HDL-c), low density lipoprotein-cholesterol (LDL-c), total cholesterol (TC) and triglycerides (TG), in people living with the human immunodeficiency virus (PLWH) on highly active antiretroviral therapy (HAART) in comparison to HIV-negative controls. Green squares represent the weight of each study in the average effect size. Horizontal lines across red squares represent the 95% confidence intervals for the point estimate. The diamonds represent the weighted average point estimate

Lipid profiles including levels of HDL-c, LDL-c, total cholesterol, and triglycerides, were also analyzed in PLWH on HAART when compared to HIV-negative controls (Fig. 6). The specific analysis of lipid profiles showing that HDL-c was significantly reduced (SMD = -1.30; 95% CI = -2.07 ‑ 0.54, z = 3.33; *p* < 0.0009) while LDL-c (SMD = 1.03; 95% CI = 0.04 ‑ 2.02, z = 2.05; *p* < 0.04) total cholesterol (SMD = 0.51; 95% CI = 0.16 ‑ 0.87, z = 2.84; *p* < 0.005) and triglycerides (SMD = 1.52; 95% CI = 0.42 ‑ 2.62, z = 2.72; *p* < 0.007) were significantly increased in PLWH on HAART in comparison to HIV-negative controls (Fig. 6). It is worth mentioning that these studies showed a substantial level of heterogeneity (I^2^ = 96%).

#### Lipid profiles in PLWH not on HAART in comparison to individuals without HIV

**Fig. 7:**
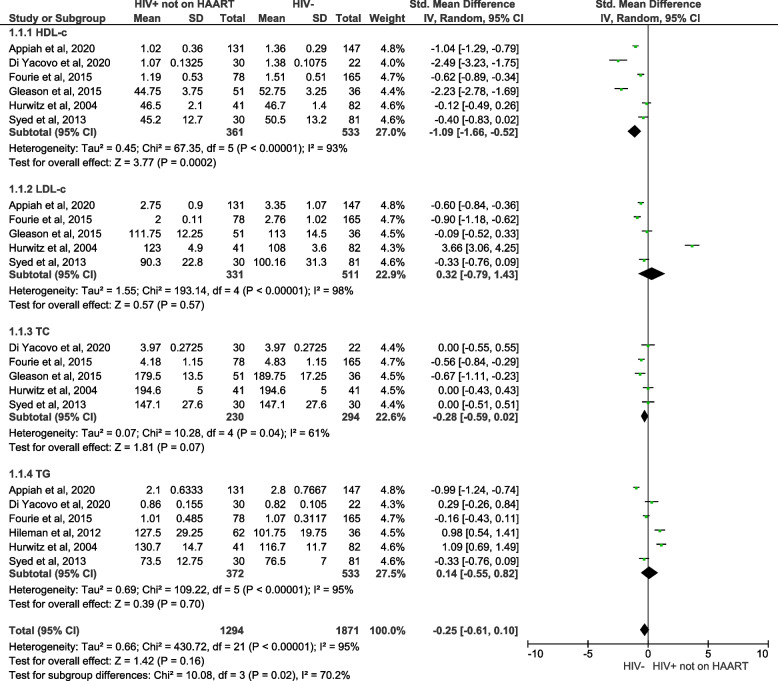
A forest plot showing outcomes of a meta-analysis for lipid profiles, including high density lipoprotein-cholesterol (HDL-c), low density lipoprotein-cholesterol (LDL-c), total cholesterol (TC) and triglycerides (TG), in people living with the human immunodeficiency virus (PLWH) not on highly active antiretroviral therapy (HAART) in comparison to negative controls or individuals without HIV. Green squares represent the weight of each study in the average effect size. Horizontal lines across red squares represent the 95% confidence intervals for the point estimate. The diamonds represent the weighted average point estimate

Lastly, this meta-analysis of pooled data aimed to assess whether HIV infection increases the levels of lipid profiles in PLWH not on HAART when compared with uninfected individuals as shown in the forest plot (Fig. 7). The pooled effect estimates displayed reduced HDL-c levels in HIV participants not on HAART when compared to HIV negative individuals (SMD = -1.09; 95% CI = -1.66- ‑0.52, z = 3.77;* p* = 0.0002). However, these studies showed a substantial level of heterogeneity (I^2^ = 93%). In addition, no association in the LDL-c (SMD = 0.32; 95% CI = -0.79‑1.43, z = 0.57; *p* = 0.57; I^2^ = 98%), TC (SMD = -0.28; 95% CI = -0.59‑0.02, z = 1.81; *p* = 0.07; I^2^ = 61%), TG (SMD = 0.14.; 95% CI = 0.55 ‑0.82, z = 0.39; *p* = 0.70; I^2^ = 95%) levels, was observed in PLWH not on HAART when compared to HIV-negative individuals.

## Discussion

Significantly contributing to the global disease burden, CVD have become a leading cause of morbidity and mortality for PLWH in the era of effective HAART [28]. It has therefore become imperative to understand the pathogenesis of CVD within PLWH, including relevant biomarkers that drive a pro-inflammatory response like hs-CRP [25]. In fact, it has been reported that hs-CRP may be a useful biomarker associated with the development of CVD-related complications in PLWH on HAART [24, 25, 58]. Despite updating the status of clinical evidence on the relevance of hs-CRP levels in PLWH, this review aimed to establish whether this pro-inflammatory marker is modulated independently in those on HAART. Importantly, beyond reporting on the levels of hs-CRP, the current review analyzed evidence on CVD-related outcomes, including lipid profiles to predict CVD risk in PLWH on HAART. To comprehend the potential influence of ethnicity and geographic locations, clinical data was additionally examined based on the world regions classified by the country where it was published. Both the systematic approach and meta-analysis were done to strengthen the reported data.

Twenty-two studies qualified for inclusion in this systematic review, with most studies (*n* = 11) coming from the African region [27, 34, 36, 38, 43–48]. The latter was expected since the sub-Saharan African region remains the epicenter of the Human immunodeficiency virus pandemic, with skyrocketing infections [3, 59]. In fact, the sub-Saharan African region is estimated to overtake high-income countries with an increased burden of noncommunicable diseases, particularly due to coronary artery disease and stroke [60, 61]. With increasing research being channeled into understanding the pathogenesis of HIV and associated complications that could be implicated in driving the development of CVD, especially the involvement of inflammation [7, 21]. Consistent with this notion, the qualitative analysis of data presented in this review clearly showed that hs-CRP levels in PLWH on HAART are significantly increased when compared to PLWH not on HAART or those without HIV (Table 1). This evidence was supported by a meta-analysis showing significantly elevated levels of hs-CRP in PLWH on HAART when compared to controls (Figs. 2 and 3). With most studies supporting this increase of hs-CRP in studies from Africa, when compared to both Europe and the United States. Further exploration using subgroup analysis did not indicate any apparent differences in statistical values, suggesting that more studies are required to assess the influence of geographical region on hs-CRP levels in PLWH on HAART. In fact, these findings that support raised levels of hs-CRP in PLWH on HAART were consistent regardless of treatment duration and were not influenced by the type of HAART regimen. This indicates that inflammation is a predominant feature in PLWH regardless of viral suppression. This hypothesis has been increasingly explored recently [21, 42], with more evidence required to understand its potential in causing the development of CVD. The findings from our analysis are consistent with results reported by Avan et al., [24] and De Luca et al., [58], where they also observed an association between the hs-CRP levels and CVD-risk in PLWH on HAART. In addition, in the Strategic Management of Antiretroviral Therapy Study (SMART), it has been shown that hs-CRP levels are associated with CVD events and all-cause mortality risk [23, 62, 63]. It's essential to note that the relationship between HIV, HAART, and inflammation is complex, and response among individuals may vary due to lifestyle and geographic locations. Furthermore, different antiretroviral drugs may have varying effects on inflammation. In cases where individuals on HAART show higher CRP levels compared to those not on HAART, a thorough investigation of factors such as medication adherence and potential concurrent health conditions is essential. Also, consistent monitoring and open discussions with healthcare providers are vital for optimizing HIV management and overall well-being.

Abnormal lipid profiles including raised levels of total cholesterol, LDL-c, and glycerides, to reduced concentrations of HDL-c have become reliable markers to evaluate CVD-risk in pathological settings [27, 64]. This explains routine measurement of lipid profiles in PLWH, including those on HAART [35, 65]. Interestingly, in our systematic review, especially in the data emanating from Africa, there was a strong correlation between elevated hs-CRP levels and abnormal lipid parameters, suggesting a potential increased risk of CVD in PLWH on HAART [27, 35, 45, 46, 50, 65, 66], which occurs regardless of the perceived benefits of treatment. Furthermore, our meta-analysis showed that HDL-c levels were significantly reduced in PLWH on HAART compared to controls (Fig. 4). Whereas the levels of LDL-c, total cholesterol, and triglycerides were significantly elevated in PLWH on HAART compared to controls (Figs. 4 and 5). Thus, the altered blood lipid status, as confirmed by evidence synthesized in this study, shows that PLWH on HAART indeed have an increased risk of developing CVD. This was in accordance with previously reported studies, which indicated that PLWH on HAART have an increased risk of developing CVD [27, 45, 65]. The increased CVD risk may relate to some of the metabolic side effects, such low bone mineral density [67], that are most pronounced in patients treated with nucleoside/nucleotide reverse transcriptase inhibitors (NRTIs) [27, 35, 45, 46, 50, 68] or protease inhibitors [47]. These findings may have clinical implications if not given enough attention. These may include implications in the development of CVD-related events such as myocardial infarction, heart failure, and peripheral artery disease because elevated levels of hs-CRP are consistent or identified in all these conditions in both PLWH and the uninfected individuals [27, 35, 69]. In addition, while the specificity of hs-CRP may be influenced by extended storage or the presence of EDTA in both serum and plasma [70–72], we are confident that these factors did not significantly impact the overarching conclusion of the present systematic review. This confidence stems from the fact that most studies included in the review reported on the serum levels of this acute phase protein (73%).

The current systematic review and meta-analysis is not without limitations. Noteworthy, most studies were composed of relatively small sample sizes; thus, the observations made remain to be explored in future studies with larger sample sizes. Another limitation is that most studies were cross-sectional rather than longitudinal, that may provide an advantage of following up on participants. There was also a high level of statistical heterogeneity among included studies. In this study the majority of studies included in the review reported on the serum levels of this acute phase protein (73%) as compared to plasma (27%). Furthermore, not all CVD-related outcomes were reported in the current analysis. However, there are also strengths of this review, which includes it being the first systematic review and meta-analysis that provides a comprehensive overview and meta-analysis of the association between hs-CRP and PLWH on HAART. Further providing much-needed information for evidence-based health care in terms of monitoring and limiting noncommunicable disease-related complications in PLWH on HAART.

## Conclusion

**Fig. 8:**
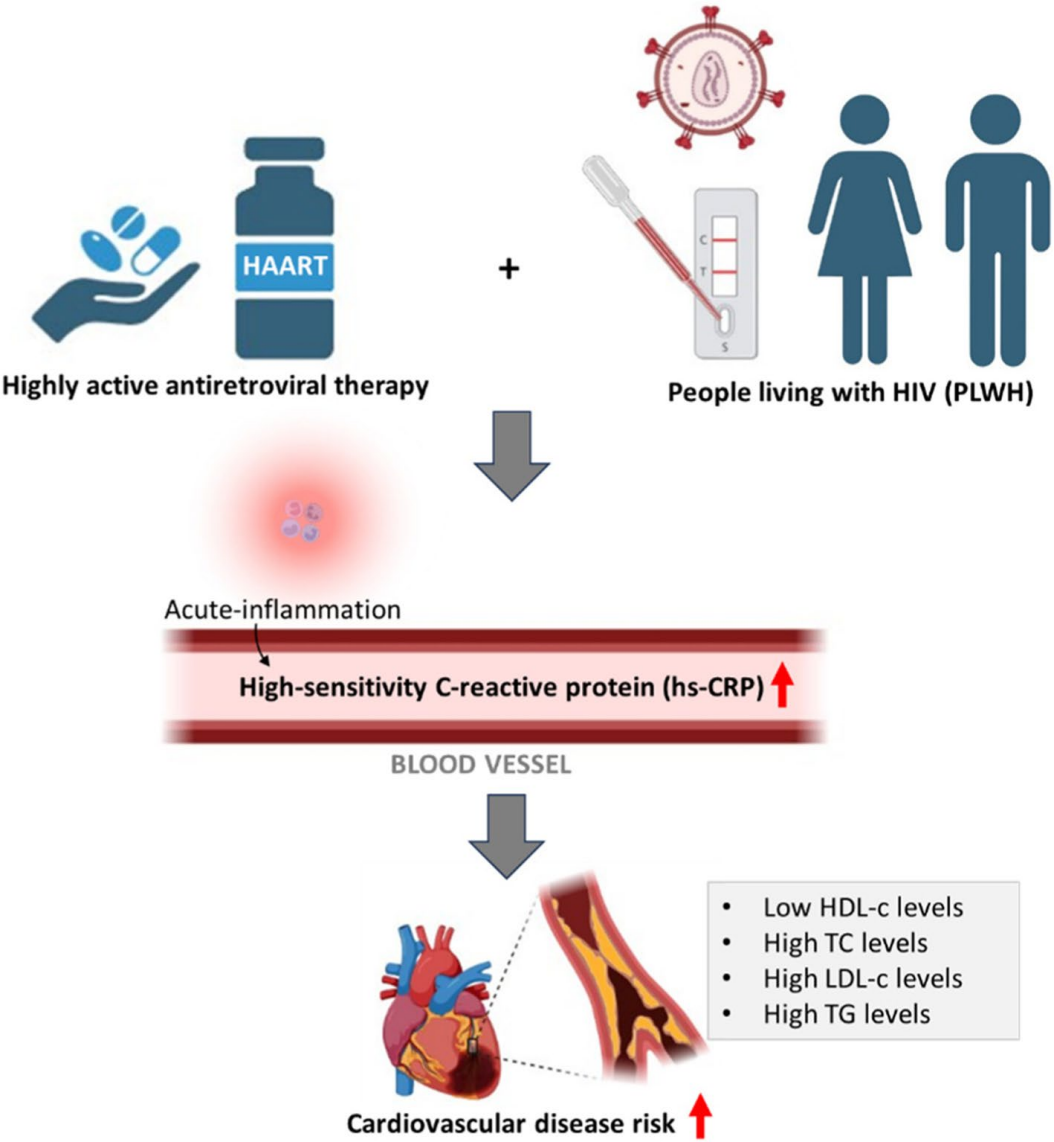
Implication of high-sensitivity C-reactive protein (hs-CRP) and lipid profiles as a potential predictors of cardiovascular disease risk in people living with HIV (PLWH) on highly active antiretroviral therapy (HAART). *HIV* Human immunodeficiency virus, *HDL-c* non-high-density lipoprotein cholesterol, *TG* triglyceride, *HDL-c* non-high-density lipoprotein cholesterol

The current systematic review and meta-analysis provide evidence that elevated levels of hs-CRP and lipid profiles are prevalent in PLWH on HAART, and this may increase the risk of CVD complications (Fig. 8). These outcomes pose challenges in policy implications on care for PLWH, especially the medical care that involves the long-term use of HAART. Addressing these challenges becomes crucial for policymakers, healthcare providers, and researchers to develop strategies that balance the long-term benefits of HAART for managing HIV with the emerging concerns related to cardiovascular risks. In addition, we motivate for large sample and longitudinal studies to be undertaken to clearly elucidate the influence of HAART use on CRP levels, lipid profiles, average age groups, follow-up duration and duration of HAART treatment and its relationship with the development of CVD in PLWH.

### Supplementary Information

**Additional file 1:** **Table S1.** Search strategy.

## Data Availability

All data generated or analysed during this study are included in this published article [and its supplementary information files].

## Abbreviations

PRISMA: Systematic Reviews and Meta-Analyses
PROSPERO: Prospective Register of Systematic Reviews
SPSS: Statistical Package for the Social Sciences
SD: Standard deviation
IQR: Interquartile range
NRTIs: Nucleoside/nucleotide reverse transcriptase inhibitors
3TC: Lamivudine
ABC: Abacavir
ATV: Atazanavir
BIC: Bictegravir
CAB: Cabotegravir
COBI or /c: Cobicistat
d4T: Stavudine
ddI: Didanosine
DMPA: Depot medroxyprogesterone acetate
DOR: Doravirine
DRV: Darunavir
DTG: Dolutegravir
EFV: Efavirenz
ETR: Etravirine
EVG: Elvitegravir
FPV: Fosamprenavir
FTC: Emtricitabine
FTR: Fostemsavir
IBA: Ibalizumab
IDV: Indinavir
LPV: Lopinavir
MVC: Maraviroc
NFV: Nelfinavir
NVP: Nevirapine
RAL: Raltegravir
RPV: Rilpivirine
RTV or /r: Ritonavir
SQV: Saquinavir
T-20: Enfuvirtide
TAF: Tenofovir alafenamide
TDF: Tenofovir disoproxil fumarate
TFV: Tenofovir
TFV-DP: Tenofovir diphosphate
Tris: Tris(hydroxymethyl)aminomethane
TMP-SMX: Trimethoprim sulfamethoxazole
TMR: Temsavir
TPV: Tipranavir
ZDV: Zidovudine
EFZ: Efavirenz
VLDL: Very low-density lipoprotein
TC: Total cholesterol
TG: Triglycerides
HDL: High density lipoprotein cholesterol
LDL: Low density lipoprotein cholesterol
hs-CRP: High sensitivity C-reactive protein
TNF-α: Tumor necrosis factor
IL-6: Interleukin
IL-8: Interleukin
BMI: Body mass index
CVD: Cardiovascular diseases
PLWH: Human immunodeficiency virus
HAART: High active antiretroviral therapy
