# Supplementary material for: High-sensitivity C-reactive protein among people living with HIV on highly active antiretroviral therapy: a systemic review and meta-analysis
Source: BMC Infect Dis. 2024 Feb 2;24:160. doi: 10.1186/s12879-024-09050-4 (PMC10838000; doi:10.1186/s12879-024-09050-4)
Supplement: Supplementary file 1 — Additional file 1: Table S1. Search strategy. [file 12879_2024_9050_MOESM1_ESM.docx]

**Supplementary Material**

***Table S1***

| **Search strategy** |
| --- |
| ("ART"[Title/Abstract] OR "antiretroviral treatment"[Title/Abstract] OR "cART"[Title/Abstract] OR "Combination Antiretroviral"[Title/Abstract] OR "HAART"[Title/Abstract] OR "Highly Active Antiretroviral Therapy"[Title/Abstract] OR "combination antiretroviral therap*"[Title/Abstract] OR "ANTIRETROVIRAL THERAPY"[Title/Abstract]) AND ("inflammat*"[Title/Abstract] OR "biomarker*"[Title/Abstract] OR "immune*"[Title/Abstract]) AND ("ABI"[Title/Abstract] OR "Ankle brachial index"[Title/Abstract] OR "Pulse Wave Velocity"[Title/Abstract] OR "PWV"[Title/Abstract] OR "FMD"[Title/Abstract] OR "Flow mediated dilation"[Title/Abstract] OR "Arterial stiffness"[Title/Abstract] OR "CIMT"[Title/Abstract] OR "thickness"[Title/Abstract] OR "Carotid intima-media"[Title/Abstract] OR "Stroke"[Title/Abstract] OR "CHD"[Title/Abstract] OR "Coronary heart disease"[Title/Abstract] OR "Mi"[Title/Abstract] OR "Myocardial infarction"[Title/Abstract] OR "cardiovascular disease"[Title/Abstract] OR "CVD"[Title/Abstract] OR "cardiovascular"[Title/Abstract]) AND ("Human immunodeficiency virus"[Title/Abstract] OR "HIV"[All Fields] OR "AIDS"[Title/Abstract] OR "Acquired immune deficiency syndrome"[Title/Abstract] OR "aids virus"[Title/Abstract] OR "acquired immune deficiency syndrome*"[Title/Abstract]) |
